# Increasing Antibiotic Resistance in *Shigella* spp. from Infected New York City Residents, New York, USA

**DOI:** 10.3201/eid2302.161203

**Published:** 2017-02

**Authors:** Kenya Murray, Vasudha Reddy, John S. Kornblum, HaeNa Waechter, Ludwin F. Chicaiza, Inessa Rubinstein, Sharon Balter, Sharon K. Greene, Sarah L. Braunstein, Jennifer L. Rakeman, Catherine M. Dentinger

**Affiliations:** Centers for Disease Control and Prevention/Council of State and Territorial Epidemiologists Applied Epidemiology Fellowship, Atlanta, Georgia, USA (K. Murray);; New York City Department of Health and Mental Hygiene, Queens, New York, USA (K. Murray, V. Reddy, J.S. Kornblum, H. Waechter, L.F. Chicaiza, I. Rubinstein, S. Balter, S.K. Greene, S.L. Bruanstein, J.L. Rakeman, C.M. Dentinger);; Centers for Disease Control and Prevention, Atlanta (C.M. Dentinger)

**Keywords:** Antimicrobial susceptibility, Shigella, MSM, azithromycin

## Abstract

Approximately 20% of *Shigella* isolates tested in New York City, New York, USA, during 2013–2015 displayed decreased azithromycin susceptibility. Case-patients were older and more frequently male and HIV infected than those with azithromycin-susceptible *Shigella* infection; 90% identified as men who have sex with men. Clinical interpretation guidelines for azithromycin resistance and outcome studies are needed.

*Shigella* bacteria are transmitted through the fecal–oral route by direct contact with an infected person, by ingestion of contaminated food or water, or by fomites. Shigellosis is associated with travel to disease-endemic areas, men who have sex with men (MSM), crowding, poverty, and attendance at childcare centers (*1*); illness is generally self-limited. Antibiotics may shorten the duration and decrease the illness severity (*2**,**3*). Because *Shigella* spp. may be resistant to ampicillin and trimethoprim/sulfamethoxazole (TMP/SMX), azithromycin and ciprofloxacin are often prescribed to treat shigellosis. In 2016, the Clinical Laboratory Standards Institute (CLSI) published MICs of azithromycin that indicated *Shigella* resistance; values are based on in vitro data and are not clinical breakpoints (*4*).

In 2013, public health laboratories in New York City (NYC), New York, USA, began testing susceptibility of *Shigella* isolates to azithromycin. We identified factors associated with infection with isolates that exhibited decreased susceptibility to azithromycin (DSA) or resistance to ciprofloxacin.

## The Study

After submission to NYC public health laboratories, representative colonies of *Shigella* isolates are identified with conventional biochemical tests and tested for susceptibility to ampicillin, cefixime, ciprofloxacin, azithromycin, and TMP/SMX using the Etest antibiotic gradient (bioMérieux, Durham, NC, USA). MICs are interpreted according to CLSI guidelines (*5*). After consultation with the Centers for Disease Control and Prevention (CDC), we defined DSA isolates as those with an MIC of azithromycin of >32 μg/mL (J. Whichard, CDC, pers. comm., 2013).

Using a standard questionnaire, we interviewed persons infected with DSA or ciprofloxacin-resistant *Shigella* isolates, diagnosed during March 22, 2013–May 31, 2015; we abstracted antibiotic use data from medical charts. *Shigella-*infected case-patients were matched to the NYC HIV Surveillance Registry (*6*). We determined neighborhood poverty level as described (*7*) and compared proportions of those infected by age group, sex, and HIV status using χ^2^ tests. To identify factors associated with DSA or ciprofloxacin-resistant *Shigella* infection and with hospitalization, we used logistic regression analysis (SAS version 9.2; SAS Institute, Cary, NC, USA).

During 26 months, 978 *Shigella* isolates were submitted; 295 were associated with an outbreak (*8*) and analyzed separately, and 683 were defined as sporadic. Among patients with sporadic infections, 129 (19%) were infected with isolates displaying DSA, and 29 (4%) were infected with ciprofloxacin-resistant isolates; 5 isolates displayed both characteristics. The median age of case-patients was 27 years (range 0–93 years); 446 (65%) were male. Nearly all infections were caused by *S. sonnei* (65%) or *S. flexneri* (34%). Antibiotic resistance of isolates was as follows: 416 (61%) to ampicillin, 10 (1%) to cefixime, 29 (4%) to ciprofloxacin, and 481 (70%) to TMP/SMX (Table 1).

**Table 1:** *Shigella* case-patient characteristics, by azithromycin or ciprofloxacin resistance, New York City, New York, USA, March 22, 2013–May 31, 2015*

Characteristic	No. (%) case-patients			
	DSA, n = 129	Ciprofloxacin resistant, n = 29	Susceptible, n = 530	Total, n = 683†
Male sex	120 (93)	22 (76)	306 (58)	446 (65)
Age, y				
0–17	3 (2)	7 (24)	254 (48)	262 (38)
18–64	119 (92)	19 (66)	261 (49)	397 (58)
>65	7 (5)	3 (10)	15 (3)	24 (4)
Race				
White	58 (45)	12 (41)	107 (20)	174 (25)
Black	39 (30)	1 (3)	75 (14)	115 (17)
Other	3 (2)	4 (14)	11 (2)	17 (2)
Unknown	29 (22)	12 (41)	337 (64)	377 (55)
Ethnicity				
Hispanic	15 (12)	3 (10)	74 (14)	91 (13)
Non-Hispanic	78(60)	15 (52)	131 (25)	222 (33)
Unknown	36(28)	11(38)	325 (61)	370 (54)
Borough				
Bronx	19 (15)	1 (3)	100 (19)	120 (18)
Brooklyn	32 (25)	7 (24)	192 (36)	230 (34)
Manhattan	61 (47)	12 (41)	119 (22)	191 (28)
Queens	16 (12)	7 (24)	111 (21)	131 (19)
Staten Island	1 (1)	2 (7)	8 (2)	11 (2)
Neighborhood poverty, %‡				
<10	23 (18)	8 (29)	87 (17)	117 (18)
10–<20	48 (38)	10 (36)	137 (27)	195 (29)
20–<30	37 (29)	7 (25)	111 (22)	152 (23)
30–100	20 (16)	3 (11)	176 (34)	199 (30)
HIV diagnosed	76 (59)	7 (24)	101 (19)	183 (27)
Antibiotic resistance by species§				
* S. sonnei*	42 (33)	23 (79)	381 (72)	443 (65)
DSA	42 (100)	3 (13)	381(100)	42 (9)
Ciprofloxacin	3 (7)	23 (100)	380 (100)§	23 (5)
Ampicillin	39 (93)	4 (17)	177 (46)	218 (49)
Cefixime	1 (1)	2 (9)	4 (1)	6 (1)
TMP/SMX	37 (88)	20 (87)	252 (66)	308 (70)
* S. flexneri*	86 (67)	5 (17)	140 (26)	230 (34)
DSA	86 (100)	1 (20)	140 (100)	86 (37)
Ciprofloxacin	1 (1)	5 (100)	139 (100)§	5 (2)
Ampicillin	79 (92)	5 (100)	109 (78)	192 (83)
Cefixime	2 (2)	1 (20)	1(1)	3 (1)
TMP/SMX	73 (85)	4 (80)	89 (64)	166 (72)

*N = 683 sporadic cases. DSA, decreased susceptibility to azithromycin; TMP/SMX, trimethoprim/sulfamethoxazole. †5 isolates resistant to ciprofloxacin also displayed DSA. ‡Percentage of residents census tract below federal poverty level, per American Community Survey, 2009–2013; 19 missing. §*S. boydii* (n = 7) and *S. dysenteriae* (n = 3) omitted.

Persons infected with DSA or ciprofloxacin-resistant *Shigella* spp. were older and more likely to be male than those with DSA- or ciprofloxacin-susceptible isolates; no association with neighborhood poverty was found. Although most infections were caused by *S. sonnei*, most isolates displaying DSA were *S. flexneri*. Isolates displaying DSA or ciprofloxacin resistance were more likely to be ampicillin- and TMP/SMX-resistant than were azithromycin- and ciprofloxacin-susceptible isolates (Tables 1, 2).

**Table 2:** Characteristics associated with azithromycin or ciprofloxacin resistance among case-patients with *Shigella flexneri* and *S.* *sonnei* infections, New York City, New York, USA, March 22, 2013–May 31, 2015*

Characteristic	No. (%) case-patients		Crude OR (95% CI)§	Adjusted OR (95% CI)§
	Resistant, n = 152†	Susceptible, n = 521‡		
Male sex	140 (92.1)	304 (58.4)	**8.33 (4.50–15.40)**	**3.27 (1.63–6.55)**
Age, y				
0–17	8 (5.3)	251 (48.2)	Referent	Referent
18–64	136 (89.5)	256 (49.1)	**16.67 (8.00–34.73)**	**7.77 (3.52–17.14)**
>65	8 (5.3)	14 (2.7)	**17.93 (5.86–54.84)**	**11.94 (3.63–39.33)**
Neighborhood poverty, %¶				
<10	30 (19.7)	85 (16.9)	Referent	Referent
10–<20	58 (38.2)	135 (26.9)	1.22 (0.73–2.04)	1.29 (0.73–2.29)
20–<30	41 (27.0)	108 (21.5)	1.08 (0.62–1.86)	1.32 (0.71–2.47)
30–100	23 (15.1)	174 (34.7)	0.38 (0.21–0.68)	0.57 (0.29–1.10)
Species				
* S. flexneri*	90 (59.2)	140 (26.7)	**3.95 (2.71–5.76)**	**1.91 (1.25–2.92)**
HIV-diagnosed				
Yes	82 (54.0)	99 (19.0)	**4.99 (3.39–7.35)**	1.44 (0.91–2.30)

*N = 683 sporadic cases. OR, odds ratio.  †*S. boydii* (n = 1) omitted. ‡*S. boydii* (n = 6) and *S. dysenteriae* (n = 3) omitted. §Values reported in bold are significant, calculated by using bivariate or multivariable (sex, age, neighborhood poverty, species, and HIV diagnosis) logistic regression. ¶Percentage of residents below federal poverty level, per American Community Survey, 2009–2013; 19 missing.

Of the 683 shigellosis case-patients, 183 (27%) had diagnosed HIV infection. Among these, 76 (42%) were infected with DSA isolates*,* and 7 (4%) were infected with ciprofloxacin-resistant isolates; 108 (59%) were infected with *S. flexneri*, 73 (40%) with *S. sonnei*, and 1 each (0.5%) with *S. boydii* and *S. dysenteriae*. Of 47 (62%) HIV-diagnosed persons with DSA *Shigella* infection, 45 (95%) identified as MSM.

Of the 153 persons with DSA- and/or ciprofloxacin-resistant *Shigella* infection, chart reviews were completed for 111 (73%). Interviews were completed for 97 (64%), and isolates of 80 (82%) of those had DSA to *Shigella*, 15 (15%) had ciprofloxacin-resistant isolates, and 2 (2%) had isolates resistant to azithromycin and ciprofloxacin. Most case-patients were male (140 [91.5%]); of 120 who completed interviews or were listed in the HIV Surveillance Registry, 102 (85%) identified as MSM. Eleven (12%) of 93 interviewed case-patients who answered the question reported international travel. All interviewees reported symptoms; most common were diarrhea (98%) and abdominal cramps (82%). Median illness duration was 7 days (range 2**–**45 days). Of 31 (32%) reported hospitalizations, 28 (90%) were infected with DSA and 3 (10%) with ciprofloxacin-resistant isolates; median duration of stay was 3 days (range 1–10 days). Twenty-five (81%) hospitalized case-patients were infected with *S. flexneri*. In a model that considered age, sex, species (*S. flexneri and S. sonnei*), HIV status, and neighborhood poverty level, only infection with *S. flexneri* was associated with hospitalization (odds ratio 4.04, 95% CI 1.46**–**11.18).

Antibiotics, most commonly ciprofloxacin, were prescribed for 114 (89%) of 128 case-patients (for whom data were available); 16 (13%) received antibiotics to which their *Shigella* isolates was not susceptible. Fifteen (17%) of 90 patients had taken antibiotics in the 4 weeks before illness onset; all were men, 10 (67%) were HIV-positive, and 12 (92%) of 13 for whom data were available were MSM.

Median illness duration for the 15 (52%) interviewed case-patients infected with ciprofloxacin-resistant *Shigella* spp. was 7 days (range 2–17 days); 3 (20%) reported hospitalization, and 5 (33%) reported recent international travel.. Five (63%) of 8 case-patients for whom data were available identified as MSM.

## Conclusions

In NYC, 19% of nonoutbreak shigellosis cases were caused by organisms with an azithromycin MIC >32 μg/mL, a much higher proportion than the national estimate of 3.8% (*9*).This finding is troubling because, when antibiotics are indicated, azithromycin is recommended (*2**,**3*).

DSA *Shigella* infection occurred almost exclusively among men, most infected with HIV. Of these, few reported travel, which suggests local acquisition. Transmission of *Shigella* spp. within networks of MSM has been described by using molecular characterization of isolates to link cases; some NYC cases are likely associated with these networks (*10*). Whether DSA *Shigella* infection results in more severe illness or in increased shedding time is unknown. Ten (59%) of 17 persons infected with ciprofloxacin-resistant *Shigella* spp. reported no exposure to disease-endemic areas (*11*). Half of non–travel-associated cases occurred among MSM, most were caused by *S. sonnei,* and few patients were hospitalized, suggesting milder illness. Strains exhibiting both ciprofloxacin resistance and DSA exist in NYC.

HIV-positive MSM may be at increased risk for acquiring infections caused by antibiotic-resistant *Shigella* spp. due to transmission-facilitating behavior (*10*) or because of increased exposure to macrolides and fluoroquinolones used to treat sexually transmitted infections, which could increase selective pressure on *Shigella* organisms (*12*). HIV infection may increase the risk of acquiring and transmitting *Shigella* infection due to increased carriage and shedding time or altered immune response (*2*).

To limit the emergence of resistance, the NYC Department of Health and Mental Hygiene and CDC recommend that antibiotics be avoided in treating *Shigella* infections except in cases of severe illness or among those at risk for systemic infection (*13**,**14*). When antibiotics are prescribed, therapy should be modified on the basis of sensitivity testing; however, without CLSI-defined clinical breakpoints for azithromycin, this process will be challenging (*4**,**14**,**15*).

We were unable to compare illness, exposures, and treatments between persons infected with susceptible versus resistant *Shigella* spp. We may have overestimated resistance if persons with resistant strains were more likely to have severe or persistent infections, seek care, and have cultures obtained. The clinical significance of resistance is not clear. Finally, molecular characterization of isolates to describe resistance mechanisms and transmission patterns was not done.

Although *Shigella* infections are generally self-limited, resistant organisms could lead to complications among those who develop systemic infection if they cannot be adequately treated. Studies of clinical breakpoints for azithromycin susceptibility and clinical outcomes are needed. In the meantime, providers should avoid treating otherwise healthy shigellosis patients with antibiotics. When antibiotics are indicated, providers should use available susceptibility results and monitor patient outcomes.
